# Supplementary material for: Mothers are more egocentric towards their own child’s bodily feelings
Source: Commun Psychol. 2023 Dec 15;1:42. doi: 10.1038/s44271-023-00038-5 (PMC7615916; doi:10.1038/s44271-023-00038-5)
Supplement: Supplementary file 1 — Supplementary Information [file 44271_2023_38_MOESM1_ESM.pdf]

Supplementary Information

Supplementary Methods

Sample sizes justification

Using G*power, we ran a post-hoc power analysis for the computed effect size of our main findings (Experiment 2 Mothers' group: EB in the 'own' group Vs EB in the 'other' group). Using an effect size of cohen's d= 0.66 (calculated based on independent t-tests, (t=2.73, p<.05, *95%CI*[0.17;1.17]; M_{EB_own}=16.36, *SE*=3.54; M_{EB_Other}=4.77, *SE*=3.53) and alpha=0.05 yielded a power of 0.76 and a critical t of 1.99. Note although the obtained power is slightly lower than 0.80 which is considered as good sensitivity, our t value is higher: t=2.73; ensuring that our sample size was coherent with the expected results. Moreover, we also ran a post-hoc sensitivity analysis using alpha=0.05 and power=0.80 and n=68 yielded a critical t of 1.66 and effect size of 0.29 which are both lower than our findings.

Supplementary Results

Experiment 1b

Correlations with social cognition measures. When exploring correlations between social cognition measures (IRI subscales, AQ10) and the EB/AB biases, only a positive correlation between the AB and IRI fantasy scale emerged ($r_{(71)}$=.37, p=.002, $p_{FDRcorrected}$=0.03), suggesting that those who show higher AB also report higher tendency to transpose themselves imaginatively into the feelings and actions of fictious characters. No other correlation survived the corrected threshold.

Comparisons between Experiment 1a and Experiment 1b samples. To explore further the variables that could explain the different results found in Exp1a and Exp1b (i.e. significant and positive AB in Exp 1a, and no significant AB in Exp1b), we conducted several independent samples tests on demographics (age and gender) and questionnaires data (IRI and AQ10). Note that for age and AQ10, Mann-Whitney U tests were conducted as the assumption of normality was violated. A threshold of p<0.01 was considered as significant, to correct for multiple comparisons. A significant difference of age between the two samples was found (U=1122, p<0.001, rB=0.662), with the sample in Exp 1a being older than sample in Exp 1b (Exp 1a: Mage=34.22, SD=10.59; Exp 1b: Mage=24.93, SD=7.79). Given results by previous studies who found an effect of age on emotional egocentric and altercentric biases (Riva et al., 2016), this could explain some of the difference observed between Experiment 1a and 1b. Moreover, significant differences were found on the IRI subscales with the sample in Exp1a scoring higher

on the fantasy scale (Exp1a: M=17.69, SD=5.18; Exp1b: M=13.79, SD=3.59; t(69)=3.507, p<0.001, d=.847), higher on the empathic concern (Exp1a: M=19.88, SD=4.66; Exp1b: M=14.37, SD=1.76; t(69)=6.049, p<0.001, d=1.46), higher on the perspective-taking subscale (Exp1a: M=18.79, SD=4.34; Exp1b: M=15.31, SD=3.42; t(69)=3.603, p<0.001, d=.870) and a trend to score lower on the personal distress subscale (Exp1a: M=11.55, SD=4.16; Exp1b: M=13.65, SD=3.16; t(69)=-2.303, p=0.024, d=-.56). However, no significant was found on the AQ10 questionnaire (U=497, p=.186, rB=-184). Note that no difference in gender was found (64% female in Exp1a vs 60% in Exp1b).

Supplementary Tables

1. <u>**Descriptives Supplementary Tables**</u>

Experiment 1

Supplementary Table 1
Descriptive statistics across the different conditions. Participants were asked to judge how pleasant was the touch they felt (Self) or saw (Other). These experiences (pleasant: cotton or Unpleasant: scourer) can either be congruent or incongruent in pleasantness.

	Self				Other			
	Pleasant		Unpleasant		Pleasant		Unpleasant	
	Incong	Cong	Incong	Cong	Incong	Cong	Incong	Cong
n	45	45	45	45	45	45	45	45
Mean	80.11	85.89	39.44	35.22	72.78	83.33	43.67	35.33
SD	12.45	10.78	20.76	17.19	16.49	11.87	21.01	16.90
Min	55.00	60.00	5.00	10.00	40.00	55.00	5.00	10.00
Max	100.0	100.0	95.00	75.00	100.0	100.0	95.00	75.00

Supplementary Table 2

Pearson's Correlations matrix between computed biases (n=45):

**Variable**	**EB**
**1. AB**	r = .48<br>p = <.001<br>BF_{10} = 44.81

AB: Altercentricity Bias; EB = Egocentricity Bias

Experiment 1b

Supplementary Table 3.A

Descriptive statistics across the different conditions. Participants were asked to judge how pleasant was the touch they felt (Self) or saw (Other). These experiences (pleasant: cotton or Unpleasant: scourer) can either be congruent or incongruent in pleasantness.

	Self				Other			
	Pleasant		Unpleasant		Pleasant		Unpleasant	
	Incong	Cong	Incong	Cong	Incong	Cong	Incong	Cong
n	30	30	30	30	30	30	30	30
Mean	82.58	84.08	23.55	25.78	61.03	67.18	37.16	28.94
SD	13.44	12.03	14.94	16.32	16.01	15.74	19.98	13.39
Min	52.75	56.00	0.75	0.00	28.00	42.75	5.00	1.25
Max	100.0	99.75	68.25	69.75	98.25	99.75	81.75	69.83

Supplementary Table 3.B

Descriptive statistics across the different unisensory experience of self (Feeling tactile only) and other (vision only).

	Feel Self		Vision Other	
	Pleasant	Unpleasant	Pleasant	Unpleasant
n	30	30	30	30
Mean	77.408	33.22	71.12	24.92
SD	16.048	17.42	15.33	16.11
Min	35.250	1.25	46.00	0.50
Max	100.0	70.75	100.0	66.00

Supplementary Table 4. Pearson's Correlations matrix between computed biases in Experiment 1b (n=30):

Variable	EB	AB	sAB
1. EB	—<br>—		
2. AB	$r = 0.48$<br>$p = .007$<br>$BF_{10} = 7.00$	—<br>—	
3. sAB	$r = 0.42$<br>$p = .02$<br>$BF_{10} = 3.00$	$r = 0.57$<br>$p = .001$<br>$BF_{10} = 38.61$	___<br>___
4. sEB	$r = 0.82$<br>$p < .001$<br>$BF_{10} = 4.19e^5$	$r = 0.45$<br>$p = .01$<br>$BF_{10} = 4.15$	$r = 0.40$<br>$p = .03$<br>$BF_{10} = 2.15$

AB: Altercentricity Bias; EB = Egocentricity Bias

Experiment 2

Mothers' Group

Supplementary Table 5.A

Descriptive statistics across the different conditions. Participants were asked to judge how pleasant was the touch they felt (Self) or saw (Other). These experiences (e.g. pleasant: cotton or Unpleasant: scourer) can either be congruent or incongruent in pleasantness. Note that mothers were paired with either their own child (Own) or other unfamiliar child (Unf).

	Judging Self								Judging Other							
	Pleasant				Unpleasant				Pleasant				Unpleasant			
	Incong		Cong		Incong		Cong		Incong		Cong		Incong		Cong	
	Own	Unf	Own	Unf	Own	Unf	Own	Unf	Own	Unf	Own	Unf	Own	Unf	Own	Unf
**n**	35	33	35	33	35	33	35	33	35	33	35	33	35	33	35	33
**Mean**	80.09	76.66	84.82	84.07	43.37	38.12	41.74	39.33	70.61	76.26	85.71	83.19	59.84	42.98	42.23	40.38
**SD**	14.68	22.44	11.86	15.58	22.57	22.00	21.93	21.28	21.50	17.65	14.35	13.97	24.80	27.57	24.05	22.79
**Min**	46.50	10.00	50.00	45.50	3.00	1.50	2.00	1.00	20.00	35.00	25.00	53.00	1.50	1.00	7.00	1.00
**Max**	100.0	100.0	100.0	100.0	85.50	85.00	80.50	85.00	100.0	100.0	100.0	100.0	100.0	100.0	95.00	87.50

Supplementary Table 5.B

Descriptive statistics across the different unisensory experience of self (Feeling tactile only) and other (vision only).

	Feeling Self				Vision Other			
	Pleasant		Unpleasant		Pleasant		Unpleasant	
	Own	Unf	Own	Unf	Own	Unf	Own	Unf
n	34	33	34	33	34	33	34	33
Mean	84.91	82.39	55.15	46.73	78.38	76.94	39.74	38.64
SD	14.78	16.68	22.51	19.35	22.38	23.10	22.95	23.70
Min	41.00	29.00	10.00	10.00	14.00	16.00	2.00	1.00
Max	100.0	100.0	97.0	89.0	100.0	100.0	82.0	95.0

Supplementary Table 6

Pearson's Correlations matrix between computed biases (n=68)

Variable	EB	AB	sAB
1. EB	—		
	—		
2. AB	$r = 0.12$	—	
	$p = .33$	—	
	$BF_{10} = 0.24$		
3. sAB	$r = 0.20$	$r = 0.43$	—
	$p = .11$	$p < 0.001$	—
	$BF_{10} = 0.52$	$BF_{10} =$ 103.28	
4.sEB	$r = 0.75$	$r = 0.17$	$r = 0.40$
	$p < .001$	$p = .18$	$p < 0.001$
	$BF_{10} = 2.10e^{10}$	$BF_{10} = 0.36$	$BF_{10} = 43.08$

AB: Altercentricity Bias; EB = Egocentricity Bias

Children group

Supplementary Table 7.A

Descriptive statistics across the different conditions. Participants were asked to judge how pleasant was the touch they felt (Self) or saw (Other). These experiences (pleasant: cotton or Unpleasant: socurer) can either be congruent or incongruent in pleasantness.
Note that children paired with either their own mother (Own) or other unfamiliar mother (Unf).

	Judging Self								Judging Other							
	Pleasant				Unpleasant				Pleasant				Unpleasant			
	Incong		Cong		Incong		Cong		Incong		Cong		Incong		Cong	
	Own	Unf	Own	Unf	Own	Unf	Own	Unf	Own	Unf	Own	Unf	Own	Unf	Own	Unf
**n**	22	23	22	23	22	23	22	23	22	23	22	23	22	23	22	23
**Mean**	9.16	8.88	9.00	8.89	6.11	5.77	5.75	5.62	6.21	6.98	8.82	8.16	6.59	6.19	4.93	5.19
**SD**	1.13	1.48	1.93	1.64	2.41	2.22	2.84	2.50	2.48	2.59	1.13	1.65	2.45	2.55	2.07	2.04
**Min**	5.50	4.00	2.00	2.50	0.00	0.50	0.00	0.00	2.00	0.50	6.00	5.00	2.00	0.00	1.00	1.00
**Max**	10.0	10.0	10.0	10.0	10.0	9.50	10.0	10.0	10.0	10.0	10.0	10.0	10.0	10.0	10.0	8.00

Supplementary Table 7.B

Descriptive statistics across the different unisensory experience of self (Feeling tactile only) and other (vision only).

	Feeling Self				Vision Other			
	Pleasant		Unpleasant		Pleasant		Unpleasant	
	Own	Unf	Own	Unf	Own	Unf	Own	Unf
n	22	23	22	23	22	23	22	23
Mean	8.93	8.75	5.45	4.91	8.96	7.90	5.02	4.35
SD	1.31	1.34	2.44	2.19	1.09	1.96	1.81	2.06
Min	6.00	5.25	0.00	0.50	7.00	2.0	2.00	0.00
Max	10.0	10.0	10.0	9.50	10.0	10.00	9.00	7.50

Supplementary Table 8

Pearson's Correlations matrix between computed biases (n=45)

Variable	EB	AB	sAB
1. EB	—		
	—		
2. AB	*r* = -0.11	—	
	p = .46	—	
	BF_{10} = 0.24		
3. sAB	*r* = -0.11	*r* = 0.08	—
	p = .46	*p* = .59	—
	BF_{10} = 0.24	BF_{10} = 0.21	
4. sEB	*r* = 0.75	*r* = -0.06	*r* = -0.09
	p < .001	*p* = .67	*p* = .57
	BF_{10} = 5.26e^{10}	BF_{10} = 0.21	BF_{10} = 0.21

AB: Altercentricity Bias; EB = Egocentricity Bias

2. Models Results' Supplementary Tables

Experiment 1

For the model results below, 'Bias' was entered as a categorical variable representing the bias type, with AB=0 and EB=1, and 'Age' in years was entered as a continuous variable. CI represent the 95% CI, and Statistic represent the t-value. P-values in bold represent significative predictor/interaction (p-value<0.05).

Model code in R was computed using the *lm4* package: lmer (Rate ~ bias + age + (1|id), data=Exp1). Tables were created using the *tab_model* function from the *sjPlot* package.

Experiment 1a

Supplementary Table 9. Model results for effects of bias type (AB vs. EB) and age on bias score (as computed from classical AB and EB score, below noted as Rate) in Experiment 1a.

a. **Classical bias.**

Predictors	*Estimates*	*std. Error*	*CI*	*Statistic*	*p*
(Intercept)	9.97	5.31	-0.59 – 20.53	1.88	0.064
bias [1]	4.44	1.82	0.82 – 8.07	2.44	**0.017**
age	-0.15	0.15	-0.44 – 0.15	-0.99	0.323
**Random Effects**					
σ^2	74.70				
$\tau_{00\ id}$	68.21				
ICC	0.48				
N_{id}	45				
Observations	90				
Marginal R^2 / Conditional R^2	0.049 / 0.503				

Experiment 1b

Supplementary Table 10. Models results for effects of bias type (AB vs. EB) and age on bias score (as computed from AB and EB score) in Experiment 1b.

a. Classical bias.

Predictors	*Estimates*	*std. Error*	*CI*	*Statistic*	*p*
(Intercept)	-6.08	6.01	-18.14 – 5.97	-1.01	0.316
Bias [1]	7.55	2.46	2.62 – 12.48	3.07	**0.003**
age	0.23	0.23	-0.22 – 0.68	1.02	0.314
**Random Effects**					
σ^2	90.73				
$\tau_{00\ id}$	44.31				
ICC	0.33				
N_{id}	30				
Observations	60				
Marginal R^2 / Conditional R^2	0.115 / 0.406				

b. **Sensory-controlled bias.**

Predictors	*Estimates*	*std. Error*	*CI*	*Statistic*	*p*

(Intercept)	-9.03	6.26	-21.57 – 3.52	-1.44	0.155
Bias [1]	18.61	2.39	13.82 – 23.39	7.79	**<0.001**
age	0.06	0.24	-0.41 – 0.54	0.27	0.786
**Random Effects**					
σ^2	85.52				
$\tau_{00\ id}$	54.99				
ICC	0.39				
N_{id}	30				
Observations	60				
Marginal R^2 / Conditional R^2	0.386 / 0.626				

Experiment 2

For the models results below, 'Bias' was entered as a categorical binary fixed factor representing the bias type, with AB=0 and EB=1, 'Pair' was entered as a categorical binary fixed factor depending on the pairing with Own=0 and Other =1, and 'Age' and 'Child age' in years were entered as continuous variables. CI represent the 95% CI, and Statistic represent the t-value.

Model code in R was computed with the following line: lmer (Rate ~ bias*Pair+Child_Age+Age+ (1|id), data=Exp2)

Mothers' sample.

Supplementary Table 11. *MLM analysis with* bias type (AB vs. EB), paired (Own vs. Other), mother's age and child's age predicting bias score (as computed from AB and EB scores).

a. **Classical bias.**

Predictors	*Estimates*	*std. Error*	*CI*	*Statistic*	*p*
(Intercept)	13.98	12.14	-10.04 – 38.00	1.15	0.252
Bias [1]	13.17	3.23	6.78 – 19.56	4.08	**<0.001**
Pair [1]	-0.33	3.53	-7.32 – 6.66	-0.09	0.927
Child Age	-0.05	0.62	-1.27 – 1.18	-0.07	0.941
age	-0.24	0.29	-0.81 – 0.33	-0.83	0.410

Bias [1] × Pair [1]	-11.50	4.63	-20.67 – -2.33	-2.48	**0.014**
**Random Effects**					
σ^2	182.33				
$\tau_{00\ id}$	24.20				
ICC	0.12				
N id	68				
Observations	136				
Marginal R^2 / Conditional R^2	0.137 / 0.238				

b. Sensory-controlled bias.

Predictors	*Estimates*	*std. Error*	*CI*	*Statistic*	*p*
(Intercept)	-0.40	18.67	-37.35 – 36.55	-0.02	0.983
Bias [1]	17.84	3.48	10.94 – 24.73	5.12	**<0.001**
Pair [1]	2.97	4.74	-6.40 – 12.35	0.63	0.531
Child Age	-0.25	0.93	-2.09 – 1.59	-0.27	0.788
age	-0.04	0.44	-0.92 – 0.83	-0.10	0.922
Bias [1] × Pair [1]	-13.88	4.96	-23.71 – -4.06	-2.80	**0.006**
**Random Effects**					
σ^2	206.28				
$\tau_{00\ id}$	155.83				
ICC	0.43				
N id	67				
Observations	134				
Marginal R^2 / Conditional R^2	0.115 / 0.496				

Experiment 2 - Children's sample:

Supplementary Table 12. *MLM analysis of* bias type (AB vs. EB), paired (Own vs. Other), child age predicting bias score (as computed from AB and EB score).

a. **Classical bias.**

Predictors	*Estimates*	*std. Error*	*CI*	*Statistic*	*p*
(Intercept)	1.15	0.96	-0.75 – 3.05	1.21	0.232
Bias [1]	2.03	0.54	0.97 – 3.10	3.79	**<0.001**
Pair [1]	-0.08	0.53	-1.14 – 0.98	-0.14	0.887
age	-0.11	0.09	-0.29 – 0.07	-1.20	0.235
Bias [1] × Pair [1]	-1.02	0.75	-2.52 – 0.47	-1.36	0.176
**Random Effects**					
σ^2	3.17				
$\tau_{00\ id}$	0.00				
N_{id}	45				
Observations	90				
Marginal R^2	0.195				

b. Sensory-controlled bias.

Predictors	*Estimates*	*std. Error*	*CI*	*Statistic*	*p*
(Intercept)	1.08	0.89	-0.70 – 2.85	1.21	0.231
Bias [1]	1.94	0.50	0.95 – 2.94	3.88	**<0.001**
Pair [1]	0.10	0.50	-0.89 – 1.09	0.21	0.836
age	-0.09	0.09	-0.26 – 0.08	-1.05	0.296
Bias [1] × Pair [1]	-0.88	0.70	-2.27 – 0.51	-1.25	0.213
**Random Effects**					
σ^2	2.75				
$\tau_{00\ id}$	0.00				
N_{id}	45				
Observations	90				
Marginal R^2	0.195				

Supplementary Results: Analyses without outliers

Note that all the codes for the analyses remained as above.

Experiment 1a.

Supplementary Table 13. *MLM analysis of* bias type (AB vs. EB) and age predicting bias score (as computed from AB and EB score) without outliers. n=44. Analysis excluding participants whose rating scores in the different conditions were more than 2.5 SD above or below the group's average.

	Rate				
Predictors	*Estimates*	*std. Error*	*CI*	*Statistic*	*p*
(Intercept)	10.54	5.39	-0.18 – 21.25	1.96	0.054
bias [1]	4.55	1.86	0.84 – 8.25	2.44	**0.017**
age	-0.16	0.15	-0.45 – 0.14	-1.07	0.288
**Random Effects**					
σ^2	76.20				
$\tau_{00\ id}$	68.29				
ICC	0.47				
N_{id}	44				
Observations	88				
Marginal R^2 / Conditional R^2	0.053 / 0.500				

Experiment 1b.

Supplementary Table 14. *MLM analysis of* bias type (AB vs. EB) and age predicting bias score (as computed from AB and EB score), without outliers. n=29.

a. **Classical bias:** Analysis excluding participants whose rating scores in the different conditions were more than 2.5 SD above or below the group's average.
b. **Sensory-controlled bias:** Analysis excluding participants whose rating scores in the different conditions were more than 2.5 SD above or below the group's average.

a. **Classical bias**

Predictors	*Estimates*	*std. Error*	*CI*	*Statistic*	*p*
(Intercept)	-6.13	6.14	-18.43 – 6.18	-1.00	0.323

Bias [1]	7.70	2.54	2.60 – 12.79	3.03	**0.004**
age	0.23	0.23	-0.23 – 0.69	1.00	0.323
**Random Effects**					
σ^2	93.64				
$\tau_{00\ id}$	46.14				
ICC	0.33				
N_{id}	29				
Observations	58				
Marginal R^2 / Conditional R^2	0.116 / 0.408				

b. **Sensory-controlled bias**

Predictors	*Estimates*	*std. Error*	*CI*	*Statistic*	*p*
(Intercept)	-9.15	6.39	-21.96 – 3.66	-1.43	0.158
Bias [1]	18.82	2.46	13.89 – 23.76	7.65	**<0.001**
age	0.06	0.24	-0.42 – 0.55	0.27	0.790
**Random Effects**					
σ^2	87.86				
$\tau_{00\ id}$	57.43				
ICC	0.40				
N_{id}	29				
Observations	58				
Marginal R^2 / Conditional R^2	0.384 / 0.627				

Experiment 2

Mothers' group

Supplementary Table 15. *MLM analysis of* bias type (AB vs. EB), paired (Own vs. Other), age and child age predicting bias score (as computed from AB and EB score), without outliers. n= 61.

a. **Classical bias:** Analysis excluding participants whose average rating scores to the Tactile only baseline unisensory pleasant condition was higher than their averaged rating in the Tactile only baseline unisensory unpleasant condition (i.e., touched by a scourer rated on average higher than touch by a cotton ball).

b. **Sensory-controlled bias** Analysis excluding participants whose average rating scores to the Tactile only baseline unisensory pleasant condition was higher than their averaged rating in the Tactile only baseline unisensory unpleasant condition (i.e., touched by a scourer rated on average higher than touch by a cotton ball).

a. **Classical bias.**

Predictors	*Estimates*	*std. Error*	*CI*	*Statistic*	*p*
(Intercept)	14.75	12.04	-9.09 – 38.59	1.23	0.223
bias [1]	15.53	3.51	8.58 – 22.49	4.42	**<0.001**
Pair [1]	-0.51	3.65	-7.74 – 6.73	-0.14	0.890
Child Age	-0.35	0.64	-1.61 – 0.91	-0.55	0.586
age	-0.19	0.29	-0.76 – 0.38	-0.67	0.507
bias [1] × Pair [1]	-13.70	4.89	-23.38 – -4.01	-2.80	**0.006**
**Random Effects**					
σ^2	185.09				
$\tau_{00\ id}$	15.75				
ICC	0.08				
N_{id}	62				
Observations	124				
Marginal R^2 / Conditional R^2	0.184 / 0.248				

b. **Sensory-controlled bias.**

Predictors	*Estimates*	*std. Error*	*CI*	*Statistic*	*p*
(Intercept)	-3.07	16.93	-36.61 – 30.48	-0.18	0.857
Bias [1]	19.18	3.85	11.55 – 26.81	4.98	**<0.001**
Pair [1]	3.01	4.60	-6.10 – 12.12	0.65	0.514
Child Age	-0.52	0.87	-2.25 – 1.20	-0.60	0.549
age	0.11	0.40	-0.68 – 0.91	0.29	0.775
Bias [1] × Pair [1]	-15.81	5.32	-26.34 – -5.28	-2.97	**0.004**
**Random Effects**					
σ^2	214.91				

$\tau_{00\ id}$	94.64
ICC	0.31
N $_{id}$	61
Observations	122
Marginal R^2 / Conditional R^2	0.148 / 0.409

Supplementary Table 16. *MLM analysis of* bias type (AB vs. EB), paired (Own vs. Other), age and child age predicting bias score (as computed from AB and EB score), without outliers. n= 64.

a. **Classical bias:** Analysis excluding participants whose rating scores in the different conditions were more than 2.5 SD above or below the group's average.
b. **Sensory-controlled bias:** Analysis excluding participants whose rating scores in the different conditions were more than 2.5 SD above or below the group's average.

a. **Classical bias.**

Predictors	*Estimates*	*std. Error*	*CI*	*Statistic*	*p*
(Intercept)	12.50	12.41	-12.06 – 37.07	1.01	0.316
Bias [1]	13.50	3.33	6.90 – 20.10	4.05	**<0.001**
Pair [1]	-0.91	3.67	-8.18 – 6.37	-0.25	0.806
Child Age	0.03	0.63	-1.22 – 1.28	0.05	0.963
age	-0.22	0.29	-0.80 – 0.37	-0.73	0.466
Bias [1] × Pair [1]	-11.40	4.83	-20.96 – -1.85	-2.36	**0.020**
**Random Effects**					
σ^2	189.00				
$\tau_{00\ id}$	23.99				
ICC	0.11				
N $_{id}$	65				
Observations	130				
Marginal R^2 / Conditional R^2	0.144 / 0.240				

b. **Sensory-controlled bias.**

Predictors	*Estimates*	*std. Error*	*CI*	*Statistic*	*p*

(Intercept)	-1.63	18.44	-38.13 – 34.88	-0.09	0.930
Bias [1]	18.09	3.50	11.16 – 25.02	5.17	**<0.001**
Pair [1]	0.85	4.76	-8.58 – 10.28	0.18	0.859
Child Age	-0.12	0.92	-1.94 – 1.69	-0.14	0.892
age	-0.02	0.43	-0.88 – 0.84	-0.04	0.965
Bias [1] × Pair [1]	-12.32	5.03	-22.27 – -2.36	-2.45	**0.016**
**Random Effects**					
σ^2	201.95				
$\tau_{00\ id}$	147.09				
ICC	0.42				
N_{id}	64				
Observations	128				
Marginal R^2 / Conditional R^2	0.134 / 0.499				

Children group.

Supplementary Table 17. *MLM analysis of* bias type (AB vs. EB), paired (Own vs. Other) and age predicting bias score (as computed from AB and EB score). n= 43.

a. **Classical bias:** Analysis excluding participants whose rating scores in the different conditions were more than 2.5 SD above or below the group's average.
b. **Sensory-controlled bias:** Analysis excluding participants whose rating scores in the different conditions were more than 2.5 SD above or below the group's average.

a. **Classical bias**

Predictors	*Estimates*	*std. Error*	*CI*	*Statistic*	*p*
(Intercept)	1.26	0.94	-0.61 – 3.12	1.34	0.183
age	-0.10	0.09	-0.29 – 0.08	-1.15	0.254
Pair	-0.21	0.53	-1.27 – 0.85	-0.40	0.693
Bias	1.77	0.54	0.71 – 2.84	3.30	**0.001**
Pair × Bias	-0.67	0.75	-2.17 – 0.82	-0.89	0.374
**Random Effects**					
σ^2	3.02				

$\tau_{00\ id}$	0.00
N_{id}	43
Observations	86
Marginal R^2	0.178

b. **Sensory-controlled bias.**

Predictors	*Estimates*	*std. Error*	*CI*	*Statistic*	*p*
(Intercept)	1.14	0.89	-0.62 – 2.90	1.29	0.201
age	-0.11	0.09	-0.28 – 0.06	-1.26	0.210
Pair	0.19	0.50	-0.81 – 1.19	0.37	0.711
Bias	1.95	0.51	0.94 – 2.96	3.85	**<0.001**
Pair × Bias	-0.76	0.71	-2.18 – 0.65	-1.08	0.284
**Random Effects**					
σ^2	2.70				
$\tau_{00\ id}$	0.00				
N_{id}	43				
Observations	86				
Marginal R^2	0.208				
