# Supplementary material for: Mothers are more egocentric towards their own child’s bodily feelings
Source: Commun Psychol. 2023 Dec 15;1:42. doi: 10.1038/s44271-023-00038-5 (PMC7615916; doi:10.1038/s44271-023-00038-5)
Supplement: Supplementary file 3 — Reporting Summary [file 44271_2023_38_MOESM3_ESM.pdf]

nature portfolio

Corresponding author(s): Louise P Kirsch

Last updated by author(s): Sep 14, 2023

Reporting Summary

Nature Portfolio wishes to improve the reproducibility of the work that we publish. This form provides structure for consistency and transparency in reporting. For further information on Nature Portfolio policies, see our Editorial Policies and the Editorial Policy Checklist.

Statistics

For all statistical analyses, confirm that the following items are present in the figure legend, table legend, main text, or Methods section.

n/a	Confirmed	
☐	☒	The exact sample size (n) for each experimental group/condition, given as a discrete number and unit of measurement
☐	☒	A statement on whether measurements were taken from distinct samples or whether the same sample was measured repeatedly
☐	☒	The statistical test(s) used AND whether they are one- or two-sided<br>*Only common tests should be described solely by name; describe more complex techniques in the Methods section.*
☐	☒	A description of all covariates tested
☐	☒	A description of any assumptions or corrections, such as tests of normality and adjustment for multiple comparisons
☐	☒	A full description of the statistical parameters including central tendency (e.g. means) or other basic estimates (e.g. regression coefficient) AND variation (e.g. standard deviation) or associated estimates of uncertainty (e.g. confidence intervals)
☐	☒	For null hypothesis testing, the test statistic (e.g. F, t, r) with confidence intervals, effect sizes, degrees of freedom and P value noted<br>*Give P values as exact values whenever suitable.*
☐	☒	For Bayesian analysis, information on the choice of priors and Markov chain Monte Carlo settings
☐	☒	For hierarchical and complex designs, identification of the appropriate level for tests and full reporting of outcomes
☐	☒	Estimates of effect sizes (e.g. Cohen's d, Pearson's r), indicating how they were calculated

Our web collection on statistics for biologists contains articles on many of the points above.

Software and code

Policy information about availability of computer code

Data collection	When the ratings were collected on computer (Exp 1b, Exp2), MATLAB and the Psychtoolbow was used to display and record participant's ratings.
Data analysis	All analyses were done with JASP and RStudio.

For manuscripts utilizing custom algorithms or software that are central to the research but not yet described in published literature, software must be made available to editors and reviewers. We strongly encourage code deposition in a community repository (e.g. GitHub). See the Nature Portfolio guidelines for submitting code & software for further information.

Data

Policy information about availability of data

All manuscripts must include a data availability statement. This statement should provide the following information, where applicable:

- Accession codes, unique identifiers, or web links for publicly available datasets
- A description of any restrictions on data availability
- For clinical datasets or third party data, please ensure that the statement adheres to our policy

De-identified data for all experiments and relevant analysis scripts in R (RStudio Team, 2022) are available on the Open Science Framework and on request to the authors (https://osf.io/xwvhg ; doi: 10.17605/OSF.IO/XWVHG).

Human research participants

Policy information about studies involving human research participants and Sex and Gender in Research.

Reporting on sex and gender	Experiment 1a: Forty-five participants (29 women and 16 men, as reported by participants)<br>Experiment 1b: Thirty participants took part in Experiment 1b and were recruited via an online SONA system (18 women and 12 men, as reported by participants)).<br>Experiment 2. Hundred thirty-six participants took part in Experiment 2 (Mothers: n=68, Children: n=68, 22 boys and 43 girls as reported by the mothers).<br>No analyses on gender were conducted as no apriori hypotheses were given.
Population characteristics	Experiment 1a: Forty-five participants (29 women and 16 men, Mage=34.72, SDage=10.68, Rangeage=20–65 years; 37 right-handed, 4 left-handed and 2 ambidextrous, as reported by participants)<br>Experiment 1b: Thirty participants took part in Experiment 1b (18 women and 12 men; Mage= 25.00, SDage=7.91, Rangeage=19–56; years, SD=7.91 years; all right-handed, as reported by participants).<br>Experiment 2. Hundred thirty-six participants took part in Experiment 2. Participants were recruited in pairs of mother and child (Mothers: n=68, Mage=43.1, SDage=4.9, Rangeage=30-57 years; Children: n=68, 22 boys and 43 girls, Mage=8.3, SDage=2.3, Rangeage=5-15 years, as reported by mothers).
Recruitment	All three experiments were conducted in accordance with the Declaration of Helsinki and were approved by the Ethics Committee of the Research Department of Clinical, Educational and Health Psychology, University College London. Written, informed consent was obtained from all participants prior to their participation. Mothers gave informed consent on behalf of their child, however, each child also gave verbally their own consent, to ensure they were comfortable with the experimental set-up.<br>Experiment 1a. Recruitment at a public event at the Royal institution, London<br>Experiment 1b. Recruitment via the Sona System of UCL.<br>Experiment 2. Hundred thirty-six participants took part during two public events: "FunFamily Day" at the Royal Institution, London; and "Self Impressions" held at the TATE Modern).
Ethics oversight	University College London

Note that full information on the approval of the study protocol must also be provided in the manuscript.

Field-specific reporting

Please select the one below that is the best fit for your research. If you are not sure, read the appropriate sections before making your selection.

☐ Life sciences ☒ Behavioural & social sciences ☐ Ecological, evolutionary & environmental sciences

For a reference copy of the document with all sections, see nature.com/documents/nr-reporting-summary-flat.pdf

Behavioural & social sciences study design

All studies must disclose on these points even when the disclosure is negative.

Study description	Quantitative Experimental
Research sample	See participants details of each experiment above.
Sampling strategy	Sample size was pseudo-random. For all experiments we aimed to recruit a minimum of 30 participants per condition (as has been done in the past by studies investigating emotional egocentricity biases and who found medium effect sizes).<br>Exp1a. Participants were recruited based on wish to participate during the Public Event<br>Exp1b. Participants were recruited via the SONA system to reach 30 participants.<br>Exp 2. Participants were recruited based on wish to participate during the Public Event
Data collection	See details in Methods section.<br>Ex: Experiment 2 followed the same design and procedure as Experiment 1b, with three exceptions: (i) Experiment 2 was conducted as a between-subject design, with mothers and children paired with either their own child / mother or other child / mother (n=23) (Figure 1.D). (ii) For the mothers, the pleasantness scale was presented on a computer (0 to 100 VAS scale), as in Experiment 1b; while children were asked to point to the relevant number in front of them on a paper-based scale, which was illustrated with cartoon faces to denote negative (unhappy) and positive (happy) affect, ranging from 0=not at all pleasant/unhappy to 10=extremely pleasant/happy. This 0-10 was used in order to facilitate ratings in children. (iii) as in Experiment 1b, two extra measures were also added as baselines: Touch only condition and Vision only condition. As in Experiment 1, mothers answered demographics questions as well as the IRI on paper.
Timing	Exp1a. June 2016; Exp 1b. November 2016. Exp2. October 2017 & April 2018

Data exclusions	Exp1. No exclusion<br>Exp 2. Given developmental findings on children's ability to take another person perspective not to develop before the age of seven and that other studies used seven years old as a standard cut-off12,19, children that were younger than seven were excluded from further analysis (n = 16). Moreover, an additional 7 children did not complete the full task, and were discarded (final children sample: n=45)
Non-participation	No participants dropped out
Randomization	For experiment 2, allocation to the group was pseudo-randomized (to balance between groups)

Reporting for specific materials, systems and methods

We require information from authors about some types of materials, experimental systems and methods used in many studies. Here, indicate whether each material, system or method listed is relevant to your study. If you are not sure if a list item applies to your research, read the appropriate section before selecting a response.

Materials & experimental systems

n/a	Involved in the study	
☒	☐	Antibodies
☒	☐	Eukaryotic cell lines
☒	☐	Palaeontology and archaeology
☒	☐	Animals and other organisms
☒	☐	Clinical data
☒	☐	Dual use research of concern

Methods

n/a	Involved in the study	
☒	☐	ChIP-seq
☒	☐	Flow cytometry
☒	☐	MRI-based neuroimaging
